# Early detection of macrovascular complications in type 2 diabetes mellitus in Medan, North Sumatera, Indonesia: A cross-sectional study

**DOI:** 10.12688/f1000research.54649.1

**Published:** 2021-08-16

**Authors:** Rina Amelia, Juliandi Harahap, Yuki Yunanda, Hendri Wijaya, Isti Ilmiati Fujiati, Zulham Yamamoto

**Affiliations:** 1 1Department of Community Medicine/Public Health, Faculty of Medicine, Universitas Sumatera Utara, Medan, North Sumatera, 20155, Indonesia; 2 Department of Pediatrics, Faculty of Medicine, Universitas Sumatera Utara, Medan, North Sumatera, 20155, Indonesia; 3 Department of Histology, Faculty of Medicine, Universitas Sumatera Utara, Medan, North Sumatera, 20155, Indonesia

**Keywords:** macrovascular complications, ankle brachial index, hydroxy vitamin D, LDL-C, triglyceride, HDL-C

## Abstract

**Background:** Macrovascular complications occur very frequently in patients with type 2 diabetes mellitus (T2DM) with a high mortality rate, due to the development of cardiovascular disease (CVD), such as stroke, atherosclerosis acceleration, and atrial fibrillation. T2DM is a significant risk factor for CVD and has become the leading cause of death. The purpose of this study was to detect the early risk of macrovascular complications by using the ankle brachial index (ABI) as a marker.

**Methods:** This study was an analytic study with a cross-sectional approach. The study population was patients with T2DM from several primary health care centers in Medan. In total, 89 subjects who met the inclusion and exclusion criteria were recruited with consecutive sampling. ABI was determined as the ratio of systolic blood pressure in the brachial artery to the posterior tibial artery after the subjects had been relaxed and felt comfortable in a supine position. Examination of vitamin D and lipid profile was derived from examination of venous blood.  Data were processed using SPSS and analyzed with one-way ANOVA.

**Results:** The study found that there was a relationship between LDL-C, triglyceride, and vitamin D (25OH-D) based on the ABI (p > 0.05).

**Conclusions:** ABI can be used for an early detection of macrovascular complications. Apart from being easy to perform, ABI was non-invasive. Some other risk factors that can also be used to assess complications and have relationships with ABI were LDL-C, triglyceride, and vitamin D (25OH-D). Complications in T2DM patients can be prevented with reasonable blood sugar control and lifestyle changes. Education and motivation need to be given to patients so that they become more independent in controlling their disease and improving their quality of life.

## Introduction

Patients with type 2 diabetes mellitus (T2DM) are at risks of short- and long-term complications. Long-term complications include macrovascular and microvascular diseases. ^1^ Macrovascular complications occur very frequently in patients with T2DM with a high mortality rate, due to the development of cardiovascular disease (CVD) such as stroke, atherosclerosis acceleration, and atrial fibrillation. T2DM is a significant risk factor for CVD and has become the leading cause of death, whereby almost 50% of the total deaths are due to this disease. ^1^ It is estimated that the mortality rate of CVD increases almost threefold in T2DM. ^2^ Besides CVD complication, peripheral arterial disease (PAD) has also become a serious condition of macrovascular complications. PAD is characterized by narrowing peripheral arteries due to atherosclerosis, and it generally occurs in the leg arteries. Many patients with T2DM are suffering from PAD, and as this disease is non-symptomatic, it is undetected. PAD is one of the T2DM complications associated with CVD. ^3– 6^ Atherosclerosis is the most common and important cause of PAD. Dyslipidemia, smoking, hypertension, and T2DM are also known as risk factors for PAD, CVD and coronary artery disease. ^7^ Ankle brachial index (ABI) is a non-invasive tool for detecting vascular status and for measuring lower leg arteries versus upper leg arterial health status. ^8^ ABI is the ratio between the lower limb (ankle) systolic blood pressure and the upper limb (upper arm) systolic blood pressure. ABI reflects vascular resistance, which explains the main factors: the blood vessel diameter, which can be narrowed either from internal factors (plaque and intimal tearing) or from external factors, such as compression by soft tissue. ^9^ ABI examination is a non-invasive gold standard measurement to detect PAD; consequently, the examination is highly recommended for patients with T2DM and others at risk of suffering from PAD. An early detection can reduce the risk of more severe complications, such as neuropathy and CVD disorders. ^5^ The purpose of this study was to detect the early risk of macrovascular complications by using the ABI as a marker.

## Methods

### Study design

This was an analytical study with a cross-sectional approach. The study had the ethical approval from the Research Ethics Commission of Universitas Sumatera Utara (Approval number No:280/KEP/USU/2020).

### Population and samples

The study population consisted of patients with T2DM in Medan. The sample size was determined using one-proportion hypothesis test with an accuracy of 10% and a significance of 95%. ^10^ A total of 89 subjects that met the inclusion and exclusion criteria were recruited with consecutive sampling technique. Patients who participated in this study were recruited from three primary health services in Medan (Medan Tuntungan, Medan Selayang, and Medan Belawan **.** The research process lasted for six months, starting from determining the patient, taking blood and laboratory examinations to completion.

Patients who regularly visited the primary health service and were willing to participate were included in this study. Patients with a history of vascular disorders before they had diabetes, leg trauma, stroke, and those with clotting blood disorders were excluded from this study. During the recruitment, selected subjects were firstly, explained about this study, the examination procedure, the process of taking blood, and explaining the discomfort experienced by the patient in the process. Next, if they agreed to join the study after they had understood the explanation about the study protocol, they were asked to sign a consent statement.

### Data collection

Most of the data collection was done between 18 ^th^ Aug 2020 and 10 ^th^ September 2020. ELISA examination, which had to be carried out in the integrated laboratory of the Faculty of Medicine of Universitas Sumatera Utara, was delayed, and could only be carried out on 10th November 2020 due to the pandemic situation that caused limited active laboratory staff with limited working hours. By early December 2020, all data had been collected and then analyzed. ABI was determined as the ratio of systolic blood pressure in the brachial artery to the posterior tibial artery. Blood pressure was measured after the subject felt relaxed and comfortable for 5 minutes in a supine position. Blood pressure was measured by using a mercury sphygmomanometer (Reister ^TM^). For measuring the blood pressure of the brachial artery, the cuff was placed at the upper arm. The systolic pressure was considered as the first occurrence of rhythmic sounds heard with stethoscope placed on the antecubital fossa. For the posterior tibial blood pressure, the cuff was placed at the ankle, the systolic pressure was considered as the first pulse was palpable on the dorsum pedis during the cuff deflation. ABI lower than 0.90 was considered abnormal, which was then classified into 0.41–0.90 as a mild to moderate decrease in blood flow, while <0.40 as a severe decrease in blood flow. ^11, 12^

Hydroxyvitamin D (25OH-D) level and lipid profile (total cholesterol, HDL-C, LDL-C, and triglyceride level) were analyzed from the venous blood samples (5 ml) drawn after 10 hours fasting. Hydroxyvitamin D (25OH-D) were measured through enzyme-linked immunosorbent assay (ELISA) with human vitamin D ELISA kit (Cat. No E1543Hu, Brand Bioassay TL). Serum for lipid profile was processed by and Auto Analyzer (Indiko Thermo Scientific™) and the total cholesterol (TC) level was measured using cholesterol oxidase method, HDL-C and LDL-C levels analyzed by enzymatic colorimetric method, ^13^ and triglyceride (TG) level was measured using the GPO–Trinded method. ^14^

Determination of the patient's nutritional status was done by using the Body Mass Index (BMI), which is defined as body weight in kilograms divided by the square of body height in meters (kg/m2), which is then adjusted to the World Health Organization classification. ^15^

### Data analysis

The data was analyzed with SPSS 22 for Windows and then shown in tables. The data under consideration was complete data; any incomplete data was deleted. We included all participants who satisfied the inclusion criteria until the minimal number of samples was reached.

The Kolmogorov Smirnov test (p > 0.05) was used to estimate the average of the normal distribution of the sample data. The normality test findings were utilized in the following analysis: parametric analysis was done if the distribution was normal; otherwise, non-parametric analysis was used. To show how ABI influenced average age, diabetes duration, blood pressure, total cholesterol, HDL-C, LDL-C, TG, and vitamin D (25OH-D) (p < 0.05), a one-way ANOVA was used. The method was then followed by least significant difference or Bonferroni testing if a significant result was obtained (p < 0.05).

## Results

Most patients were female (69 subjects, 77.5%), and from the age group of 46–55 years (37 subjects, 41.6%). Normal nutritional status was noted in 44 subjects (49.4%). Based on the duration of illness, more than half of the subjects (47 subjects, 52.8%) had T2DM for 1–5 years ( Table 1).

**Table 1.** Basic characteristics of type 2 diabetes mellitus patients.

Characteristics	Number of patients (n = 89)	Percentage (%)
Gender Male Female	20 69	22.5 77.5
Age, years (mean, SD)	55.2	8.9
Age group <36 years 36-45 years 46-55 years 56-65 years >65 years	1 12 37 29 10	1.1 13.5 41.6 32.6 11.2
Nutritional Status (BMI = kg/m ^2^) Underweight (<18.5) Normal (18.5-24-9) Overweight (25.0-29.9) Grade 1Obese (30.0-34.9) Grade 2 Obese (35.0-39.9)	3 44 25 14 3	3.4 49.4 28.1 15.7 3.4
Duration of illness, years (mean, SD) <1 year 1-5 years 6-10 years >10 years	4.4 18 47 16 8	4.3 20.2 52.8 18.0 9.0

Table 2 shows that 34 (38.2%) patients were in the borderline PAD category, meaning that the peripheral circulation had begun to be disrupted but not included as having PAD. In contrast, 26 patients (29.2%) had mild PAD.

**Table 2.** Ankle brachial index (ABI) classification in patients with type 2 diabetes mellitus.

ABI classification	Number of patients (n = 89)	Percentage (%)
Normal	29	32.6
Borderline	34	38.2
Mild PAD	26	29.2

Table 3 indicated that there were differences in average levels of LDL-C, TG and vitamin D (25OH-D), based on ABI (p < 0.05). However, age, duration of illness, blood pressure, TC, and HDL-C did not show this difference (p > 0.05). It was concluded that there was a relationship between LDL-C, TG, and vitamin D (25OH-D) based on ABI classification.

**Table 3.** Ankle brachial index (ABI) classification in patients with type 2 diabetes mellitus.

Variables	ABI Classification						p
	Normal		Borderline		Mild peripheral arterial disease		
	Mean	SD	Mean	SD	Mean	SD	
Age	55.6	9.0	53.2	4.8	49.0	12.0	0.225
Duration of diabetes	4.6	4.4	3.3	3.6	2.3	2.3	0.405
Blood Pressure (systole) (mmHg)	149.5	21.1	167.5	31.9	156.7	16.3	0.353
Total cholesterol (mg/dl)	219.5	42.1	220	40.1	249.3	49.2	0.950
HDL-C (mg/dl)	46.6	11.8	44.7	9.2	53.3	12.8	0.750
LDL-C (mg/dl)	126.6	35.8	124.0	30.2	150.0	13.0	0.0325 ^*^
Triglycerides (mg/dl)	242.7	127.1	253.8	67.0	350.3	162.9	0.0406 ^*^
Vitamin D (25OH-D) (ng/ml)	16.5	0,26	16.4	0,26	25.0	0,21	0.0393 ^*^

## Discussion

Diabetes with vascular inflammation has become a risk factor for atherothrombotic diseases, such as PAD. ^16^ A low ABI value reflects atherosclerosis in the vessel wall, resulting in a decrease of perfusion and arterial circulation to the distal extremities. ^17^ This reduction in perfusion is usually characterized by loss of peripheral pulses, intermittent claudication, and complicated infection of the legs. ^18^ The pathogenesis of atherosclerosis in PAD is the same as that of the coronary arteries. The lesion in the segment creates stenosis or occlusion that usually takes place in the large or medium-sized vessels. The development of these lesions become atherosclerotic plaques with calcium build-up, thin tunica media, destruction of muscle and elastic fibers, and fragmentation of the elastic internal lamina; and thrombus consisting of platelets and fibrin may happen. ^19^

Hypertension and diabetes increase the risk of the development of macrovascular and microvascular complications. ^7, 20^ According to a study by Hiramoto (2014), after 30 months of observation, the ABI value decreased in patients with T2DM by 20.7%, with 5% of the patients showing PAD symptoms. ^19^ The factors causing the decrease in ABI were influenced by age, gender, high HbA1c, high serum creatinine, high LDL-C, and retinopathy. ^21^

*Dyslipidemia* is a metabolic disorder associated with diabetes. It is characterized by a spectrum of quantitative and qualitative changes in lipids and lipoproteins, including hypertriglyceridemia, decreased concentrations of high-cholesterol lipoprotein (HDL), and elevated LDL. ^22^ Various studies have proven that the risk factors for cardiovascular disease and death from coronary heart disease are directly related to the concentration of cholesterol in the blood. ^23^ Hypercholesterolemia can directly cause endothelial dysfunction through the production of reactive oxygen species, which converts low-density lipoprotein (LDL) into oxidized low-density lipoprotein (ox-LDL). ^24^ LDL-C and TG are markers that have been used to assess the occurrence of atherosclerosis (atherogenic dyslipidemia). ^25, 26^ Atherogenic index of plasma, on the other hand, is an instrument that has been used to assess the occurrence of atherosclerosis by using TG level as a marker. ^27, 28^ Ultimately, small dense LDL-C leads to an increased risk for the development of CVD. ^29– 31^

This study indicated that there was no relationship between the length of illness and age with ABI. The results were not in line with other studies that showed that age and length of illness affecting ABI, and that this risk increased in patients aged ≥50 years and a history of diabetes-associated atherosclerosis. ^7^ Complications such as uncontrolled blood sugar levels, i.e., random blood sugar level ≥ 200 mg/dL, and fasting blood sugar level ≥ 126 mg/dL occur in T2DM patients within an average period of 5–10 years. ^32^ The low ABI is influenced by irregular consumption of anti-hyperglycemic drugs, irregular physical activity, irregular foot care, and irregularity in the T2DM diet. ^33^

This study shows that there was relationship between vitamin D and ABI. Vitamin D is associated with arterial atherosclerosis, which underlies an increased risk of CVD, ^34– 36^ and there is a strong association between vitamin D deficiency and the prevalence and severity of PAD, ^37^ however, other studies have demonstrated that serum levels of Vitamin D are not associated with arterial stiffness or PAD. ^36^ Previous studies have reported that vitamin D has a preventive role in patients with CVD. ^39^

This vitamin inhibits the renin–angiotensin–aldosterone system and modulates macrophage activity and cytokine production. ^35^ T2DM and vascular inflammation have become risk markers and even risk factors for atherothrombotic disease, including PAD. T2DM increases the process of atheroma formation. There is an increase in histamine levels in plasma and cells in patients with T2DM and PAD, which can lead to an increased endothelial permeability. ^40^ The next process is the migration of T-lymphocytes into the intima and the increase in the secretion and the activation of cytokinesis. Monocytes/macrophages ingest oxidized LDL molecules and turn them into foam cells, where the accumulation of these cells will form fatty streaks, which are the precursors of atheroma. Atheroma plaque will become unstable because endothelial cells in patients with T2DM secrete cytokines that inhibit collagen production by smooth muscle cells of the blood vessel. Metalloproteinases are also released by these inflammatory cells, which can destroy the collagen fibrous cap plaque atheroma, increasing the tendency for plaque rupture and thrombus formation. ^16^ Systemic inflammation has also been evident to increase insulin resistance. T2DM is an inflammatory condition, and vitamin D may reduce insulin resistance by reducing the risk of inflammation.

There is an association between low vitamin D levels and T2DM, and impaired glucose tolerance. ^41^ In populations with vitamin D deficiency and impaired glucose tolerance and T2DM, vitamin D supplementation can improve insulin secretion, glucose tolerance, and decrease HbA1c levels. The provision of vitamin D supplementation in healthy adults with this deficiency has shown to improve insulin sensitivity by 60%, which is better than rosiglitazone and other metformin therapy. ^42^

The abnormalities in the function of endothelial cells and vascular smooth muscle and the tendency to thrombosis are affected by atherosclerosis and its complications. Endothelial cells can regulate blood vessel function and structure because of the strategic anatomical position between the blood vessel walls and blood flow. Under normal circumstances, many active substances are synthesized and released by endothelial cells to maintain blood vessel homeostasis and to regulate blood flow and nutrients to the tissues while preventing thrombosis and leukocyte diapedesis. ^43^

Individuals with low serum vitamin D levels have an increased risk of dyslipidemia, insulin resistance, and T2DM. The effect of vitamin D on the improvement of T2DM can be directly obtained through insulin action. Vitamin D enhances insulin response to glucose transport by stimulating insulin receptor expression in peripheral tissues. Moreover, vitamin D improves the level and the integrity of lamellae in the aortic medium tunica and prevents fragmentation of the aortic elastic fibers. Vitamin D supplementation in adult patients with T2DM significantly improves lipid profiles. Therefore, it can be concluded that fat metabolism disorders are caused by vitamin D deficiency. ^44^

The limitation of this study was that it did not distinguish patients with a history of smoking from non-smoking individuals. The results of other studies also say that a history of or smoking affects blood vessels because it causes atherosclerosis in blood vessels that contributing significantly to the ABI value, and smoking can significantly increase DM patients experiencing diabetic peripheral neuropathy. ^45^

## Conclusions

Early detection of macrovascular complications is vital to prevent morbidity and mortality in T2DM patients. ABI can be used for the early detection of macrovascular complications. Besides being simple and non-invasive, the ABI is also entirely accurate for detecting macrovascular complications in T2DM patients. The results demonstrated that there was a correlation between the ABI value and the average LDL-C level, triglycerides, and vitamin D.

## Data availability

### Underlying data

Figshare: Laboratory Result of type 2 DM Patients, https://doi.org/10.6084/m9.figshare.14915724.v1. ^46^

This project contains the following underlying data:

• Data file (physical examination results, patient characteristics, and laboratory results of patients (BGL, HbA1c, Total cholesterol, LDL-C, HDL-C, TG, Vitamin D)

Data are available under the terms of the Creative Commons Zero “No rights reserved” data waiver (CC0 1.0 Public domain dedication).
