# Supplementary material for: Early detection of macrovascular complications in type 2 diabetes mellitus in Medan, North Sumatera, Indonesia: A cross-sectional study
Source: F1000Res. 2021 Aug 16;10:808. [Version 1] doi: 10.12688/f1000research.54649.1 (PMC8411276; doi:10.12688/f1000research.54649.1)
Supplement: Peer review history

# Peer review: Reviewer response for version 1

**DOI:** 10.5256/f1000research.58153.r91990

**Published:** 2021-09-01

**Authors:** Hari Kusnanto

**Affiliations:** 1 Department of Family and Community Medicine, Faculty of Medicine, Public Health and Nursing, Gadjah Mada University, Yogyakarta, Indonesia

This study is simple yet very powerful in advocating the use of Ankle Brachial Index as a risk measurement for peripheral arterial disease as a manifestation of macrovascular problem. The predictive capability of ABI to measure the risk for peripheral arterial disease is also consistent with the level of LDL-cholesterol and triglycerides known to be risk factors for cardiovascular diseases. Of particular interest, which should be emphasized in the discussion is concerning the positive association between the level of vitamin D and the level of peripheral arterial disease. This is a very important finding now when many people consume high dose of vitamin D to prevent severe COVID-19. Evidence-based explanation of the negative effect of higher level of vitamin D need some more elaboration.

Is the work clearly and accurately presented and does it cite the current literature?

Yes

If applicable, is the statistical analysis and its interpretation appropriate?

Partly

Are all the source data underlying the results available to ensure full reproducibility?

Yes

Is the study design appropriate and is the work technically sound?

Yes

Are the conclusions drawn adequately supported by the results?

Yes

Are sufficient details of methods and analysis provided to allow replication by others?

Yes

Reviewer Expertise:

My areas of research is epidemiology as applied to family medicine

I confirm that I have read this submission and believe that I have an appropriate level of expertise to confirm that it is of an acceptable scientific standard.

# Peer review: Reviewer response for version 1

**DOI:** 10.5256/f1000research.58153.r91992

**Published:** 2021-08-31

**Authors:** Mohd Adzim Khalili Rohin

**Affiliations:** 1 School of Nutrition and Dietetics, Faculty of Health Sciences, Sultan Zainal Abidin University, Kuala Terengganu, Malaysia

- The manuscripts could be improved if the authors include detailed information in methods with sufficient data and supporting evidence in results and discussion sections.
- The introduction needs to be justified, with limited evidence to show the magnitude of the problem.
- The conclusion could be more concise and better worded study implications.
- Language-wise is good.
- Limited references were used in supporting the findings. See Silva *et al.* (2017[ref 1]),  Angellotti *et al. *(2017 ^2^) and Palomer *et al. *(2008 ^3^).

Is the work clearly and accurately presented and does it cite the current literature?

Yes

If applicable, is the statistical analysis and its interpretation appropriate?

Yes

Are all the source data underlying the results available to ensure full reproducibility?

Yes

Is the study design appropriate and is the work technically sound?

Yes

Are the conclusions drawn adequately supported by the results?

Partly

Are sufficient details of methods and analysis provided to allow replication by others?

Yes

Reviewer Expertise:

Community Nutrition, Functional Foods & Nutraceuticals

I confirm that I have read this submission and believe that I have an appropriate level of expertise to confirm that it is of an acceptable scientific standard.

## References

[1] : Risk factors and complications in type 2 diabetes outpatients.*Rev Assoc Med Bras (1992)*.2017;63(7) : 10.1590/1806-9282.63.07.621621-62710.1590/1806-9282.63.07.62128977088

[2] : The Role of Vitamin D in the Prevention of Type 2 Diabetes: To D or Not to D?. *Endocrinology*.2017;158(7) : 10.1210/en.2017-002652013-202110.1210/en.2017-00265PMC550521928486616

[3] : Role of vitamin D in the pathogenesis of type 2 diabetes mellitus.*Diabetes Obes Metab*.2008;10(3) : 10.1111/j.1463-1326.2007.00710.x185-9710.1111/j.1463-1326.2007.00710.x18269634
